# Embedding qualitative research in randomised controlled trials to improve recruitment: findings from two recruitment optimisation studies of orthopaedic surgical trials

**DOI:** 10.1186/s13063-021-05420-4

**Published:** 2021-07-17

**Authors:** Arabella Scantlebury, Catriona McDaid, Stephen Brealey, Elizabeth Cook, Hemant Sharma, Arun Ranganathan, Joy Adamson, on behalf of the ACTIVE and PRESTO study teams

**Affiliations:** 1 Department of Health Sciences, York Trials Unit, University of York, York, YO10 5DD UK; 2 Hull University Teaching Hospitals NHS Foundation Trust, Hull, HU16 5JQ UK; 3 Bart’s Health NHS Trust, The Royal London Hospital, Whitechapel Road, London, E1 1BB UK

**Keywords:** Process evaluation, Recruitment, Retention, Randomised controlled trial, Orthopaedic, Surgery, Qualitative

## Abstract

**Background:**

Recruitment of patients is one of the main challenges when designing and conducting randomised controlled trials (RCTs). Trials of rare injuries or those that include surgical interventions pose added challenges due to the small number of potentially eligible patients and issues with patient preferences and surgeon equipoise. We explore key issues to consider when recruiting to orthopaedic surgical trials from the perspective of staff and patients with the aim of informing the development of strategies to improve recruitment in future research.

**Design:**

Two qualitative process evaluations of a UK-wide orthopaedic surgical RCT (ACTIVE) and mixed methods randomised feasibility study (PRESTO). Qualitative semi-structured interviews were conducted and data was analysed thematically.

**Setting:**

NHS secondary care organisations throughout the UK. Interviews were undertaken via telephone.

**Participants:**

Thirty-seven health professionals including UK-based spinal and orthopaedic surgeons and individuals involved in recruitment to the ACTIVE and PRESTO studies (e.g. research nurses, surgeons, physiotherapists). Twenty-two patients including patients who agreed to participate in the ACTIVE and PRESTO studies (n=15) and patients that declined participation in the ACTIVE study (n=7) were interviewed.

**Results:**

We used a mixed methods systematic review of recruiting patients to randomised controlled trials as a framework for reporting and analysing our findings. Our findings mapped onto those identified in the systematic review and highlighted the importance of equipoise, randomisation, communication, patient’s circumstances, altruism and trust in clinical and research teams. Our findings also emphasised the importance of considering how eligibility criteria are operationalised and the impact of complex patient pathways when recruiting to surgical trials. In particular, the influence of health professionals, who are not involved in trial recruitment, on patients’ treatment preferences by suggesting they would receive a certain treatment ahead of recruitment consultations should not be underestimated.

**Conclusions:**

A wealth of evidence exploring factors affecting recruitment to randomised controlled trials exists. A methodological shift is now required to ensure that this evidence is used by all those involved in recruitment and to ensure that existing knowledge is translated into methods for optimising recruitment to future trials.

**Trial registration:**

ACTIVE: (ISRCTN98152560). Registered on 06/03/2018. PRESTO: (ISRCTN12094890). Registered on 22/02/2018,

**Supplementary Information:**

The online version contains supplementary material available at 10.1186/s13063-021-05420-4.

## Background

Participant recruitment is a key challenge for randomised controlled trials (RCTs). Failing to reach recruitment targets can have significant cost implications if funding is stopped or extensions are required and trials that do not recruit to target are underpowered to detect an effect [1]. Trials of surgical interventions, especially those that compare surgical and non-surgical interventions face recruitment challenges, due to the irreversibility of surgical treatments and issues with surgeon equipoise [2–5]. The stakes are particularly high for trials of rare fractures or injuries, where there are a limited number of eligible patients and so there is an added impetus to recruit as many eligible patients as possible.

There is growing evidence on the barriers and facilitators to surgical trial recruitment [3, 6]. A mixed methods systematic review synthesised the evidence on recruitment to surgical trials from the patient perspective [1]. The review, which included 34 papers (11 quantitative and 21 qualitative), concluded that whether a patient chooses to participate or not in a RCT is influenced by multiple inter-related factors such as patients’ perceptions of the risk and benefits of participation and their treatment preferences. The authors recommended that future trials consider a patient-centred approach to trial recruitment that tailors information to the individual patient. For recruitment to be effective, it is also important to take stock of staff’s experiences of recruitment to ensure that future strategies for improving recruitment consider not only what is important to patients, but the practicalities of recruiting patients in a complex healthcare system [1].

We present the findings of two qualitative process evaluations undertaken during the PRESTO (Pragmatic Randomised Evaluation of Stable Thoracolumbar fracture Treatment Outcomes) feasibility study [7] and ACTIVE (Articular type C pilon fracture Trial comparing Internal Versus External fixation) trial’s internal pilot. The main a priori research question for both process evaluations was how to best optimise recruitment by exploring patients, surgeons and local recruiting staffs’ views and experiences of the interventions and trial processes. In this paper, we draw on the work of Phelps et al. [1] and use the findings from our two process evaluations to highlight how recruitment to future orthopaedic RCTs may be improved from the perspective of trial recruiters and patients.

## Methods

### Design

Two qualitative evaluations of orthopaedic surgical trials were undertaken and consisted of interviews with patients who agreed to take part in the ACTIVE or PRESTO studies, interviews with patients who declined participation and interviews with ACTIVE and PRESTO trial recruiters (surgeons, physiotherapists, research nurses). Interviews with surgeons from across the UK (who were not part of recruiting teams) were conducted during PRESTO as one of the main aims of the study was to understand the current treatment of stable thoracolumbar fractures. Data collection took place during the PRESTO study’s 12-month recruitment phase and the ACTIVE study’s 12-month internal pilot recruitment phase (Table 1).

**Table 1:** ACTIVE and PRESTO study details

	ACTIVE	PRESTO
Objectives	To investigate the clinical and cost-effectiveness of internal plate fixation versus external fine wire fixation for the management of type C pilon fractures of the distal tibia.	To establish whether it is feasible to deliver a trial comparing surgical fixation to initial non-surgical management for patients with a stable thoracolumbar fracture without spinal cord injury.
Design	Parallel randomised controlled trial. A 12-month internal pilot with associated qualitative study to assess recruitment and provide guidance on optimising trial processes.	A feasibility study consisting of three elements: a randomised external feasibility study, a national survey of surgeons and a qualitative study.
Trial interventions	Arm 1: Internal fixation: ‘Locking’ plate fixation with screws. Arm 2: External frame fixation: limited open reduction and articular fixation using screws and fine wire fixator.	Arm 1: Surgical fixation. Either open spinal surgery or minimally invasive surgery as per current UK surgical treatment. Arm 2: Non-surgical management. Mobilisation in a brace or mobilisation without a brace.
Primary outcome	Disability Rating Index at 12 months post-randomisation.	Recruitment rate, defined as the proportion of eligible participants who are randomised.
Trial status and registration	Ongoing (ISRCTN98152560)	Completed (ISRCTN12094890)
Ethical approval	Approved by Yorkshire and The Humber – Bradford Leeds REC on 13^th^ February 2018 (REC reference 18/YH/0014).	Approved by North East – Newcastle and North Tyneside Research Ethics Committee (REC) on 20^th^ March 2018 (REC reference 18/NE/0008).
Funder	NIHR Health Technology Assessment Programme (HTA 15/130/84)	NIHR Health Technology Assessment Programme (HTA 15/154/07)

### Sampling and recruitment

To obtain maximum variation, we sought to purposively sample patients on the basis of age, gender, recruitment site and treatment received. Given the lower than anticipated recruitment to the main PRESTO and ACTIVE studies and process evaluations, we adopted a convenience sampling frame with selection based on those who agreed to interview. In both studies, all patients, irrespective of whether they agreed to participate in the trial were invited to take part in the qualitative study and were provided with a separate participant information sheet and consent form. A contact details form was also completed and transferred to the qualitative team for those that declined to participate in the ACTIVE or PRESTO studies. Informed consent was obtained prior to each interview.

For the ACTIVE trial, initially, we aimed to only interview staff who had been directly involved in recruiting patients. Due to the staggered opening of sites and lower than anticipated recruitment of patients, we also invited staff at sites open for, but just prior to recruitment. ACTIVE trial recruiters were invited to interview via email.

For PRESTO, trial recruiters from the 3 participating sites were interviewed. In addition, snowballing techniques were used to ensure that a wide range of surgeons from across the UK were interviewed. This approach is appropriate when the number of experts in the field is relatively small and so many surgeons are known to each other [8]. However, we utilised a range of additional recruitment strategies to ensure interviewees represented a range of geographical locations, specialties (orthopaedics, neurosurgeons) and grades (consultants, registrars). We asked clinical co-applicants to forward recruitment emails to surgical colleagues, both in and outside of PRESTO sites; advertised for participants through the British Association of Spine Surgeons (BASS) newsletter and through a regional network of spinal surgeons; and by sending an invitation email to those who expressed an interest in the qualitative study through the PRESTO survey (n=19).

### Participants

Thirty-seven surgeons and trial recruiters (research nurses, research physios) were interviewed during the PRESTO (n=19) and ACTIVE (n=18) process evaluations. Surgeons across both studies represented different grades (registrars and consultants) and specialties (orthopaedics, neurosurgery). Additionally, 15 patients who agreed to take part in the PRESTO (n=5) and ACTIVE (n=10) process evaluations were interviewed. ACTIVE interviewees represented a range of genders and treatment allocations. However, all but one of the PRESTO patient interviewees were male and received surgical fixation. Interviews were conducted with 7 patients that declined to participate in the ACTIVE trial, no patients that declined to participate in PRESTO were interviewed.

### Semi-structured interviews with patients, surgeons and trial recruiters

All interviews were semi-structured, were conducted via telephone and followed topic guides that were developed by the research team and clinical co-applicants. Interviewees were asked a range of questions, which aimed to explore how patients were approached to take part in the two studies, trial procedures and current treatment. Further details of the topics covered during interviews can be found in Additional files 1, 2, 3, 4, 5 and 6.

### Analysis

Data collection and analysis were undertaken by AS, a health services researcher with no clinical training or prior experience of research in orthopaedic surgery. All interviews and consultation recordings were audio-recorded and transcribed verbatim. For both studies, an initial thematic analysis was undertaken of all interviews and consultation recordings for each individual study [9]. Themes were largely deductive and derived from study topic guides, whilst also allowing for emergent themes. Analyses for both studies was also influenced by ‘initial impressions’ which were recorded immediately in an excel spreadsheet and involved documenting key issues under a priori headings that were devised by the research team, topic guide and clinical co-applicants. Documenting initial impressions allowed for early familiarisation and preliminary coding of our data and provided a mechanism for highlighting key issues with recruitment to the Trial Management Group and recruitment sites throughout data collection. A secondary analysis was then conducted where the findings from both studies were mapped onto the themes identified in Phelps’ systematic review [1], with particular attention given to identifying themes relating to optimising recruitment.

## Results

First, we present the findings of both studies according to the three main themes that were identified by Phelps et al.’s [1] as influencing recruitment and retention to surgical trials—*making sense*, *weighing up* and *trust*. Given the significant amount of evidence that already exists around these themes [1, 10–15], we present our findings using summary tables to highlight the key issues that were identified during ACTIVE and PRESTO. We then provide a descriptive account of ‘eligibility criteria’ and ‘patient pathways’, two themes that were identified as influencing recruitment and retention during ACTIVE and PRESTO which are not featured in Phelps et al.’s review [1].

### Making sense

#### Randomisation

For patients to agree to participate in an RCT, they first need to ‘make sense’ of randomisation and the influence it has on their treatment and involvement in their care (Table 2). Randomisation was a new concept for the majority of patients, which when described using trial jargon made the concept difficult to understand. In the most part, patients recalled randomisation; however, many had undergone significant trauma and so were heavily medicated when approached to take part in the study. As a result, patients were concerned that they lacked capacity at the time of recruitment and felt this restricted their ability to understand what was being proposed and make informed decisions. However, trial recruiters described various methods that were used during PRESTO to ensure capacity, for example, patients were asked to reiterate study aims before providing their consent.

**Table 2:** Key findings relating to randomisation

Randomisation		
Sub-theme	Description	Exemplar quote
Understanding randomisation	For most patients randomisation was a new concept	“They told me it was 50/50 whether they would give me surgery, or I would be in a brace, and it was chosen quite randomly by the computer. No human influence on that, at all. They obviously said that my care and everything would be exactly the same as if I wasn’t on the trial.” (PRESTO patient interview, accepter)
Trial jargon	Across both trials, staff felt patients equated randomisation to being experimented on and to remove their choice and decision making around treatment. Using the analogy of a toss of a coin was considered flippant and unprofessional – ACTIVE patients felt gambling metaphors undermined the severity of their injury whilst trial recruiters felt that describing something as random implies it is intrinsically bad or inferior. Using computer-based terminology invoked fear with some patients who were reluctant to be randomised due to a mistrust in computers and technology – patients wanted treatment to be based on an informed choice by a qualified and experienced person. Clear explanations of terms such as randomisation, are more likely to promote transparency, understanding and acceptance. Some ACTIVE trial recruiters thought that they should not use the word randomisation and in some cases avoided discussing that patients would be allocated to treatments by a computer as they were aware of the potential negative implications of this terminology on recruitment.	“I think when you try to say randomised and talk about the computer allocating it, people worry. It frightens people, and I wonder if maybe my experience of that is within a study that general has recruits who are in an older age bracket and may be a little bit more unsure of technology and computers and want the doctor to make their decision”. (ACTIVE Staff Interview) “It did seem kind of not as professional. Like, “We’re going to do this really serious thing on you”, this was serious and obviously all operations and procedures like that, it must cost a fortune, and for them to basically just, it was almost like it came down to “flip the coin”” (ACTIVE Patient interview, accepter)
Recall of randomisation	The severity of PRESTO patient’s injuries, the medication they were prescribed and presence of other injuries led to the capacity of these patients and whether they made informed decisions about their treatment and trial participation to be questioned. Many PRESTO patients found it difficult to engage with study materials and/or were unable to remember details of recruitment and randomisation.	“But it probably would have been better if it was explained when I had someone with me, because as I say, I was very sleepy. I’d had lots of morphine, and I think I could just about keep my eyes open when they were talking to me.” (PRESTO patient, accepter)
	PRESTO staff did not consider the patient’s capacity to be an issue as they felt obtaining informed consent and determining capacity during recruitment mirrored the processes used in routine practice.	“I don’t think that (capacity is) a problem most of the time. If you think about it, to do anything you have to get informed consent, whether it’s a trial or not. If I operate on someone, I have to get their consent. If I’ve discussed surgery or not surgery, implicitly they’re consenting to have not surgery if we pursue bed rest. Things can always change. I mean you can operate further down the line.” (PRESTO staff interview,)

#### Communication

How the trial is communicated to patients is also a critical factor influencing a patient’s decision to take part in a RCT. In both studies, the perceived quality of information sheets, information about treatment and recovery and when patients are approached to take part were pivotal to ensuring effective communication and influenced whether patients chose to take part (Table 3).

**Table 3:** Key findings relating to communicating research to patients

Communication		
Sub-theme	Description	Exemplar quote
Information sheets	Patients in both trials were generally satisfied with the written information they received. Some PRESTO trial recruiters thought study documentation was appropriate, others felt the volume of information could overwhelm patients, particularly given the nature of their injuries. Shortening study documents and adding links to online information were suggested.	“I knew I was part of the trial but I really couldn’t remember what any of the details were, so it was nice to know what I’d actually agreed to.” (PRESTO patient interview, accepter).
Information about treatment and recovery	In both trials, there were a number of patients that felt they did not receive enough information about study interventions and that information was not presented equitably. Junior doctors were deemed unable to answer questions and consultants too busy. Particular emphasis was thought to have been placed on the risks of surgery rather than on the impact of the interventions on daily life and return to work. Patient perceived there to be an imbalance between the time spent discussing the trial and treatment options. PRESTO patients in particular felt they did not receive enough information about recovery.	“I came away thinking it was a spandex suit basically. Honestly that’s all I knew about it. I think maybe a picture of the potential thing you’d have to wear and also how long on average you would probably wear these things for, like just a bit more…yeah basic things like does it affect what you can wear? They were definitely answered in a way by the 3 members of the team but I can’t really recall their answers so I don’t think they were that good.” *(*PRESTO patient interview, accepter)
Reflecting and considering trial information	In both studies, participants found speaking to staff on multiple occasions and being given time to consider their participation and read study documentation beneficial. For one PRESTO patient, having the time to consider their participation led to them changing their mind and agreeing to take part.	“I said no at first. I had only just come in that day, and I was, you know, not with it still, but then I changed my mind.” (PRESTO patient interview, accepter)

#### Equipoise

Patients and trial recruiters provided various examples of situations where equipoise had been either deliberately, or unintentionally undermined (Table 4).

**Table 4:** Key findings relating to equipoise

Equipoise		
Sub-theme	Description	Exemplar quote
Patient understanding of equipoise	Emphasising that clinical teams consider both treatments to be appropriate for the patient, are effective, routinely provided and lead to good outcomes is important.	“The main point that we put across to the patient when we discuss randomisation is that at the present moment, the debate regarding the surgery or conservative options is pretty equal among the way consultants have been practising all across the UK. So there has been no preferred treatment for either of those options. And that is what we were trying to find, and detailing the options as both an equally suggestive and successful treatment options then makes the patients more receptive to the idea of randomisation.” (PRESTO staff interview)
*Communicating uncertainty*	Disagreement as to what is considered a stable thoracolumbar fracture, concerns that study treatments are not comparable and the perceived inherent opinionated nature of surgeons meant that surgeons found it difficult to discuss the uncertainty surrounding treatment options and culminated in subtle attempts to undermine equipoise. PRESTO patients gave examples of surgeons expressing preferences for the opposing treatment to what they had been allocated post-randomisation. ACTIVE patients sensed relief, annoyance and disappointment from staff depending on their allocation – they felt relieved when their allocation matched the preference of the recruiter.	“I don’t think [Surgeon] was very happy because I was going through a trial and he wanted to put the external frame on. Then they put the plates in. […] you’re doing this trial, and he wanted to put the cage thing on my leg. I was like, “Why doesn’t he do it then, if he thought it was going to work better?”[…] I can’t really remember, but he was really off with me though”. (ACTIVE patient interview, accepter)
*Remaining neutral*	In both studies, patients felt staff conveyed preferences for specific treatments. For example, some PRESTO patients described either not being given a choice of treatment, or explained how after being allocated to one treatment the surgeon stated that they would preferred them to have the other. Patients challenged staff neutrality by asking them what treatment they would routinely recommend.	“My partner who was there all the time was given a choice of having the surgery for my back or a brace and he said “No, they said surgery.” (PRESTO patient interview, accepter)
Variation in routine practice	In general, PRESTO surgeons were positive about the need for a trial and saw it as crucial to address variation in practice. It was suggested that there is a proportion of surgeons at every hospital who would not be willing to randomise or be part of a trial—some surgeons not involved in recruitment to PRESTO felt it was inappropriate to ever operate on stable fractures, others had different views.	“I don’t think there’s much variability at all for stable fractures, except for a few places in the country, nobody operates for these patients. If I asked everyone, except for five or six or ten surgeons in the UK they operate for stable fractures, otherwise people don’t operate. So those ten surgeons you can identify first in the UK and then run the trials using those surgeons then you can finish the trial. Otherwise you don’t want to start the trial and then take two or three years to recruit the patients, if you still want to run this trial.” (PRESTO staff interview)

### Weighing up

Patients’ decisions to participate in RCTs are underpinned by being in equipoise and their understanding of randomisation. However, these decisions also require patients to trade off various perceived benefits of participation, such as altruism against treatment preferences, personal circumstances and the perceived risks of randomisation (Table 5).

**Table 5:** Factors that patients consider when ‘weighing up’ their participation in a RCT

Weighing up		
Sub-theme	Description	Exemplar quote
Altruism	Patients valued and understood the need for clinical research and participated for reasons such as wanting to advance medical knowledge and/or help others. Only a small number of staff used altruism in recruitment consultations.	“It seemed to me that the frame was the best option and I didn’t really want to take that risk, but then I felt bad, not bad but – because how on earth is research going to develop if people don’t take part in research projects” (ACTIVE patient interview, decliner)
‘Doing what’s best for me’	PRESTO patients described participating to “sticking two fingers” to family members who had strong treatment preferences and because they thought they would receive better care if they were part of a trial. Others seemed indifferent towards participation and claimed ‘I’m going to be lying on my back anyway, so what the heck’	“They were explaining to me that it would be helping other people, but I said actually at the time I was more interested in what was best for me, rather than what was best for other people. But I was happy to go ahead with the trial and that I did want an operation. And if they did come back and say that it would have been the brace and not the operation I would have said that I didn’t want to be part of the trial.” (PRESTO patient interview, accepter)
Personal circumstances/daily life	Of those that declined to participate in ACTIVE many based their decision on the perceived impact of the intervention on their daily life. For example, the ability to mobilise and/or wear normal clothing. Comfort, ease of commuting, positive experiences of previous surgery and specific concerns about compliance and the impact of the brace on daily life (PRESTO) were cited.	“I said I would rather have the operation. I know It sounds…I had an operation on my lower back ten years ago which was completely different. I researched – because I’m a single dad – I mean, my daughter’s with her other dad now who lives abroad for her six week holiday, which is actually a good time. That was planned anyway. You know, I didn’t want to be limited because I am a single dad, and I run a business. So, it’s like I wanted to recover as soon as possible”. (PRESTO patient interview, accepter).
Perceived recovery time	Perceived recovery time and return to work were key concerns across both trials. Both ACTIVE and PRESTO patients that we interviewed had preferences for surgery due to a perceived quicker recovery time.	“If anything had been quicker, I just wanted to get out of there, there and then […] If anything had been quicker I would’ve been like that, ‘Oh, do it. Get me out of here’, really” (ACTIVE patient interview, accepter)
Friends, relatives and other external sources	Opinions and vicarious experiences of friends, relatives and other non-professionals and patient’s own research influenced patient decision making. In ACTIVE, people were influenced by others that had received either treatment for other fractures.	“[My wife] was Googling everything up under the sun about it for me as well and she came to the same decision as me. She thought it was a good idea that I’d chosen that, the external frame method because it just did seem like hacking into my leg was a bit too much” (ACTIVE patient interview, decliner)
The impact of interventions on body image	During ACTIVE, concerns about scarring from internal fixation and how others may react to the external frame were prevalent. Recruiters reported trying to address body image related concerns by involving limb reconstruction nurses in recruitment consultations, where possible	“I just couldn’t see how anybody could possibly live with this barbaric contraption. It looks like a two-year old’s pedal bike just wrapped around my leg” (ACTIVE patient interview, accepter).
Addressing treatment preferences	For the majority of patients, preferences were considered changeable and examples of patients changing their mind were provided. Trial recruiters dedicated part of recruitment consultations to addressing this by discussing the pros and cons of each treatment and tailoring information to the concerns of individuals. However, staff explained that there will always be a subset of patients with strong, unchangeable preferences. Those which based decisions on personal (e.g. children, fear) rather than clinical reasons were considered particularly difficult to change. Sometimes patients feel that they have expert knowledge of their own body and so believe there must be a ‘best’ option for any given individual. These patients often want to maintain control of their own health and do not want others making decisions for them. Staff and patients provided examples of situations where participation depended on allocation aligning with the treatment preferences of patients. Some patients claimed they would have withdrawn if they had not been randomised to their preferred treatment.	“I said, oh I’m pretty much having surgery and he kind of went “well no”. He took me through the options. So they were very clear the way they described it and it was interesting because there was a lot of debate because my sister was there and my sister was calling my mother and my sister and mother wanted me to have surgery because they knew somebody who had a brace for scoliosis and said it was extremely uncomfortable and their entire life, you know, the period they were in a brace was pretty frustrating and so yeah there was definitely….from the three members of the medical team in the room and [name] was quite good at arguing, not arguing, but basically giving the other opinions to my sister and my mother who were kind of blasting me in one ear.” (PRESTO patient interview, accepter).
Minimising risk of randomisation	Some associate randomisation with a loss of autonomy. Both patients and clinicians described how making patients aware that clinicians have the final say in patient’s treatment and that irrespective of their allocation they will receive the clinically appropriate treatment was important along with emphasising a patient’s right to withdraw. In PRESTO, patients were informed they could switch treatments if deemed clinically necessary during recruitment for those allocated to surgery or follow-up for those allocated a brace. In ACTIVE patients had been ‘put off’ participation by a treatment being portrayed as more risky – this was perceived to undermine equipoise by suggesting a surgeon feels one treatment is better than another.	“We are guided by the computer, purely because both the treatment options are correct.” That makes them feel a little bit more in control, like if you say that one is better than the other, “Why am I being randomised then?” When you say to them that both the options are pretty equal, then they are happy and they know that there is always that opportunity to cross over, because you do say to them that, “If during the follow up process that we are treating you with a brace, if we believe that things aren’t going in the right way, then trial, no trial, we’ll switch you to whatever’s appropriate.” That is the good thing that the patients recognise, that their treatment is carrying on as it should be, whichever way the trial goes, whether you go in the trial or you don’t go in the trial, everything put together. I think that should reassure them that we are more trying to treat them appropriately rather than the trial. The trial comes second.” (PRESTO staff interview).

### Trust

If patients are to take part in a surgical trial, they need to feel confident and have trust in their surgeon and research team. When discussing how to optimise recruitment, it was considered crucial by the trial team to think about who is involved in recruitment consultations (Table 6). Whilst there was agreement that recruitment consultations should involve senior clinicians and the research team, the practicalities of involving both teams in large numbers of recruitment consultations were acknowledged, with some sites opting for the research team and clinicians to approach patients independently to fit around conflicting work schedules and time constraints.

Ensuring clinical teams are engaged throughout research studies was also considered important by both trial recruiters and patients not only for ensuring the trust of patients but to maximise recruitment, particularly in studies of rare conditions where there is pressure to recruit every eligible patient.

**Table 6:** Key findings relating to trust

Trust		
Sub-theme	Description	Exemplar quote
Who should be involved in recruiting patients	The majority of staff felt that recruitment should be a joint venture involving a senior clinician and the research team to instil confidence and trust in the trial and ensure patient expectations are met. Senior clinicians can build cases to support both treatments, provide reassurance about them being routinely provided and communicate the grey areas and complexities surrounding treatment. Consultants were considered best placed to challenge patients with strong preferences, particularly when based on information received from clinicians in other specialties. Research staff are crucial for overcoming equipoise issues and communicating study details as this is part of their daily role. Patients were aware when recruiters did not appear knowledgeable about interventions. Avoiding key terminology was perceived to cause uncertainty and undermine confidence and trust.	“The pros are that the clinician has a very in-depth understanding and is used to presenting information about those two treatment options. In think the negative, sometimes can be in relation to knowledge of how a research trial works and the potential pitfalls of presenting information in a certain way that might lead the patient one way or the other, and how careful we need to be with that. Also, to make the patient feel very much not under pressure to join the trial. I think that’s something that, perhaps the research team is a bit better at, having more experience in that area, So I think there are pros and cons, and that obviously is clinician dependent and research team member dependent. Certainly what we found worked really well here, was to have the clinician present the clinical parts of it so that the patient knows that the clinical information is coming from a clinician who would be treating them. Then for the research team to explain participation, withdrawal procedures and all that kind of stuff.” (PRESTO staff interview)
Practicalities of ‘joint’ consultations	Time constraints, concerns about surgeon equipoise and availability meant the practicalities of involving surgeons and research staff at the same time at all consultations is challenging. To overcome this, at some sites, surgeons and research staff held separate conversations with patients about treatments and the study respectively.	“So I speak to them first. I tell them what it entails if they’re interested to be involved in the trial. If they are interested then I will let the research nurse know, who will also go through all the details of the trial.” (PRESTO staff interview)
Engagement of clinical teams	The central role of clinical teams in recruitment and checking whether patients are eligible means ensuring their continuous engagement is crucial in surgical trials. PRESTO staff were concerned that PIs would lose enthusiasm over time and that reduced engagement could negatively affect recruitment.	“It was to be a study that lasts a long time and there can be periods where there’s not much recruitment, the concern is then that people get a bit bored and disengaged with it, and I think that’s a little but what’s happened to be honest. And I think if there was a trial that was going on for four, five years, by this point, people had already started to lose interest.” (PRESTO staff interview)

### Patient pathways

Patients are often admitted via complex referral pathways and as a result, may come into contact with clinicians from a range of specialties, depending on the specific injury (emergency department (ED), neurosurgery, orthopaedics) or from referring hospitals prior to being approached to take part in orthopaedic surgical trials. In PRESTO, the different ways that patients with thoracolumbar fractures could be referred to sites was a particular issue, with one site receiving referrals from up to 15 hospitals. The reduced awareness of on-going research studies, issues with equipoise and different treatment preferences of staff that are not involved in recruitment can mean that patients have multiple conversations about the treatment they are ‘likely’ to receive before official recruitment consultations and may lead to distrust in research and trial processes and/or patient’s being ‘biased’ towards specific treatments. The time between transfer of patients from local hospitals to Major Trauma Centres or hubs can mean that by the time patients are approached to take part in a research study they may no longer meet the eligibility criteria for the specific trial, for example they have already started to heal and in some cases receive non-operative treatment such as braces for stabilisation.

*We generally want to see them within a week of them sustaining the injury and a few times it has not been possible to arrange transport from the local hospital up to our centre, and that has caused patients to miss the reasonable timeframe that we would like to follow these patients, for example if they reach five to six weeks following the injury by the time to come to the clinic, then it is a moot point to then discuss surgical or operative options. Because conservatively they have reached that point in their treatment, where the fracture could have reasonable been expected to heal and we wouldn’t be doing justice to either the patient or the trial to then discuss the two separate options for them. (PRESTO staff interview 06)*

Communication between patients and specialties or clinicians that were not involved in trial recruitment was thought to have caused some patients across both studies to develop treatment preferences and view randomisation as unacceptable. Insufficient buy-in from clinical colleagues was seen by PRESTO recruiters to exacerbate this issue. Whilst attempts were made during PRESTO to increase engagement of neurosurgeons through multiple departmental presentations the difficulties of asking for a culture change ‘ahead of evidence’ was acknowledged particularly given the issues with surgical equipoise, the comparatively low levels of research activity in neurosurgical departments and the fact that neurosurgeons routinely provide the perceived less resource-intensive option of conservative management.

*The neurosurgery team here are not the most helpful. (Laughter) I don’t know whether that's because we haven’t communicated the study very well or whether that’s just their mentality to research. We have decided that we will do a presentation to them again to make sure that it’s not a fault on our side. You’ll probably hear (name of staff) talk about this as well, but we did notice a couple of the patients that we were screening were being reviewed or presented in A&E and then neurosurgery registrars would be going down to review them on the request of A&E doctors. Then they would discharge them from there, so they weren’t getting admitted, which meant that those patients who have been told that they don’t need to be managed with an- they essentially just get discharged with a brace. A couple of those patients we, potentially, could have included in PRESTO, but because we missed them due to- I guess the registrars were completely unaware of the trial. (PRESTO staff interview, 10)*

There were particular concerns amongst recruiters in both trials that treatment conversations between patients and EDs lead to patients being ‘biased’ towards a specific treatment. In addition to undermining equipoise, one patient described how receiving a call to return to hospital to discuss the possibility of surgery and participate in a trial after being discharged with either no or conservative treatment caused anxiety and provided a poor standard of care. Engaging ED doctors ahead of a full trial was considered a particular challenge given the pressure on ED to treat patients quickly to avoid affecting performance targets and the fact that patients are often admitted ‘out of hours’ which was perceived to increase the likelihood of patients being seen by doctors with a lower awareness of research studies based in other departments. High staff turnover, particularly of surgical registrars meant that Junior Doctors were also considered to be prone to inadvertently influencing patient expectations and be unaware of ongoing research protocols.

*Unfortunately these fractures come in late at night or early in the morning and they’re already seen by a junior member of the team. The junior staff change over so frequently, they’re not aware of the trial, and they just go by…..meaning for stable fractures don’t fix, for unstable fractures, fix, because that’s what you’ve been taught traditionally. Once they’ve told them in A&E that they don’t need an operation, because this trial is all about stable fractures, then it is quite difficult to convince the patients otherwise. (PRESTO staff interview)*

Variation in current practice and strong treatment preferences meant that the influence of ‘local logistics’, having strict eligibility criteria and carefully considering how to approach sites in future trials was considered crucial. Presenting a future trial, planned protocol and eligibility criteria at key clinical meetings was also suggested as a means to secure buy-in from the clinical community. Importance was placed on having centralised centres for recruitment with it considered unfeasible for referring hospitals to be involved in recruitment. Engaging staff from other specialties at recruiting sites was also considered important. Despite this, there were doubts as to the feasibility and implications on time and resources of involving multiple specialties in recruitment to a future trial, a number of suggestions were proposed and are displayed in Table 7.

**Table 7:** Involving multiple specialties in recruitment to future orthopaedic surgical trials

Suggestion for involving multiple specialties in the recruitment of patients to future orthopaedic RCTs	
Encourage inter-specialty collaboration through associate principal investigators (PIs)	Appointing clinical trainees as associate PIs may encourage inter-specialty collaboration, optimise recruitment and provide study support. Trial teams should provide manuals to support associate PIs and encourage professional benefits to individuals for adopting these roles such as Continuing Professional Development (CPD) (ACTIVE, PRESTO)
Name clinicians from specialties not involved in recruitment on delegation logs.	Ensuring surgeons from other key specialties and particularly registrars are on delegation logs to ‘intercept the pathway’ and account for the fact that treatment decisions are often made quickly. (PRESTO)
Raise awareness of trials through regular presentations and posters at trial sites.	To account for staffing changes and junior doctor rotation, regular presentations and briefings about studies that are open for recruitment are important. (PRESTO) Trial teams should distribute posters to study sites for display in all departments where potentially eligible patients are assessed and/or admitted. (ACTIVE)
Use technology to encourage communication between clinical and research teams.	Creating a WhatsApp group for clinical and research staff to improve communication and ensure that patients are approached to take part in studies as soon as is appropriate after admission. (PRESTO)
Provide opportunities for research staff to discuss potentially eligible patients.	Research staff attending handover meetings in person or receiving daily handovers from specialties involved in recruitment is important to ensure no potentially eligible patients are missed. (PRESTO)
Encourage the involvement of on-call staff in recruitment	Ensure on-call staff are aware of study recruitment criteria and have access to the study team’s contact details (ACTIVE)
Ensure mechanisms for communication between key specialties and PIs at trial sites are established and known.	Asking specialties to directly call the PI or study team as soon as a patient is identified, so they can be screened and an appointment arranged immediately. (PRESTO)
Involve the Emergency Department (ED) in recruitment	Involving ED staff in recruitment and ensuring ED is being screened daily is important to avoid missing potentially eligible patients. Out of hours support for research staff, is particularly important for units that ‘encroach on ED’ to accommodate for out of hours admissions. Having an open clinic for patients to be booked into from ED may be of benefit. (PRESTO)
Use electronic systems where possible to identify eligible patients	Sites should consider searching electronic systems where these are available to identify all eligible patients (ACTIVE)

### Eligibility criteria

Difficulties operationalising eligibility criteria were reported across both trials and were largely due to clinical teams interpreting clinical criteria differently. For example, in PRESTO, there was a lack of consensus as to what constitutes a stable thoracolumbar fracture, whilst in ACTIVE there was disagreement as to the definition of an open fracture. Other factors which were perceived to affect the number of eligible patients during PRESTO included: difficulties obtaining consent and completing study documentation for patients for whom English is not their first language; mental health; frailty; patients living in a different area to the treating hospital and so wanting to be followed up locally and patients at high risk of infection or of encountering wound problems and wound breakdown. It was however suggested that staff may be ‘hiding behind personal biases’ rather than excluding patients for ‘objective reasons’. Despite having screened approximately 80% of the predicted numbers of patients overall in the PRESTO study, surgeons reported that the target population was smaller than anticipated—the majority of patients were either considered ‘very stable’ or ‘very unstable’; therefore, far fewer patients met the eligibility criteria than was predicted.

*I guess, perhaps, that will be much more useful to look at, at the end, when we see the trends really clearly and you can say, “Well, okay, this was a huge number of people we excluded for this reason. Was that actually justified, in terms of the science that we have out there at the moment, to say, “This group of patients should have been managed as they were, and excluded”? (PRESTO, staff interview)*

## Discussion

This paper reports the findings of two qualitative studies that were conducted during a large, multi-centre, UK-wide RCT’s internal pilot phase (ACTIVE) and a mixed methods feasibility study’s (PRESTO) recruitment period. We used a recent mixed methods review of patients’ experiences of being recruited to surgical trials as a framework for reporting our findings [1]. Our data mapped onto the key themes identified by Phelps [1] and suggest that a patient’s decision to take part in a RCT is determined by how they can ‘*make sense*’ of the trial and the impact of participation on patient care and daily life; a process of ‘*weighing up*’ where patients trade off the risks and benefits associated with participation and ‘*trust*’ in the treating clinicians and the research team.

We also identified two additional factors that affect recruitment, which are not reported by Phelps—the impact of patient pathways and how eligibility criteria are interpreted and applied by clinical staff [1]. Although recognised as barriers to trial recruitment [10, 11, 15], they have been given less attention in the recruitment optimisation literature than factors such as equipoise, randomisation and communication. The relative lack of attention paid to patient pathways may in part be due to the differential impact this will have across trials. The complexity of patient pathways will vary according to the surgical area of interest and is likely to be site specific. Some approaches to addressing the issues would potentially require a large investment of resources for both trials units and site staff. For example, many of the strategies for addressing issues arising from complex patient pathways that we propose in Table 7 would require trial recruiters to ensure that staff from multiple specialties are aware of a trial and are prepared to support the identification and recruitment of patients throughout a trial’s recruitment period. The complexities of this should not be underestimated, particularly when considering: the implications of staff turnover and part-time workers; the number of sites and specialties that can be involved in recruitment, particularly in large national trials such as ACTIVE (n= 30), the difficulties of balancing recruitment against routine clinical work; and the number of RCTs and research studies that can be on-going at any given site. In addition to these practical issues, problems with equipoise and the strong and divided opinions of clinicians around current treatment often underpinned many of the problems we identified with patient pathways and operationalising eligibility criteria.

During PRESTO, despite trial recruiters using well established and routinely used methods for determining whether a patient has capacity to provide informed consent, some PRESTO patients perceived themselves to lack capacity at the time of recruitment. The discrepancy between how staff and patients perceived the informed consent process during the PRESTO trial suggests that current methods for establishing capacity may need to be improved. This finding speaks to a larger ongoing discussion relating to the consent process in research. Much of this discussion has focussed on moving away from our current emphasis on a single moment of consent to an ongoing consent dialogue with research participants [16, 17]. However, this is difficult to achieve in clinical practice and has significant implications for how we currently approach consent within trials as it would require all stakeholders (including researchers and ethics committees) to adapt to what might potentially become a less well-defined process. The CONSULT study [18] is an on-going NIHR funded study which is focussed on improving the inclusion of adults lacking capacity in research through a decision support intervention to help family members to make decisions about research. Whilst this could equally be applied to potential trial participants for whom lack of capacity is transient, it is not the only solution. A collective effort is required to develop multiple strategies and/or interventions to improve and optimise consent processes which consider the needs of different populations and contexts.

The mixed methods systematic review conducted by Phelps and a recent Cochrane qualitative evidence synthesis provide two comprehensive evaluations of the evidence on factors affecting recruitment to RCTs from the perspective of trial participants [1, 15]. When combined with our findings, which incorporated the perspective of both patients and trial recruiters, it is clear that the same issues relating to equipoise, how randomisation is communicated and the impact of patient preferences are repeatedly reported as barriers to recruitment across a range of RCTs. It could therefore be argued that we have reached a saturation point of evidence exploring barriers and facilitators to trial recruitment. We do not dispute the value of including recruitment optimisation interventions within RCTs, especially bespoke solutions which target trial and site-specific issues in detail, e.g. QuinteT (Qualitative research integrated within trials) [19–21]. However, we suggest that future work focusses on ensuring that existing evidence is disseminated, applied and used to inform the design of process evaluations and recruitment to future RCTs if we are to avoid further research waste [22]. More specifically, there is a need for trials units to ‘lead by example’ and ensure that their staff are up to date on the current evidence on recruitment optimisation and directly apply this to site set-up, development of trial documentation (e.g. participant information sheets), trial design and recruitment. Training such as ‘Granule’ [23] should be completed by all those involved in recruitment and research teams to maximise the efficiency of site initiation visits and to ensure that study documentation and recruitment consultations are in line with the latest evidence. Lastly, developing methods that apply existing evidence, but require minimal resources, should be a methodological priority if we are to make use of the existing evidence base and optimise recruitment to future trials.

### Strengths and limitations

We used a recent mixed methods systematic review [1] as a framework for reporting our findings, which has enabled us to go beyond a descriptive account and place our findings in the context of existing evidence. Our findings are based on data that was collected from patients who agreed and declined to participate in a trial and the views of surgeons and trial recruiters. This enabled us to obtain a more detailed understanding of the complexities surrounding trial recruitment than would have been possible had we have only explored this issue from the perspective of one stakeholder group. Trial recruiters across both studies expressed uncertainties surrounding the appropriateness of interviewing patient decliners despite this being established practice. In the ACTIVE study, we were able to mitigate against this and provide reassurance through site initiation visits and additional, separate follow up phone calls with sites about the qualitative study and this may account for the discrepancy in recruitment of patient decliners between the two trials. As such, we would recommend undertaking this additional preliminary work when designing future process evaluations.

## Conclusion

This study has highlighted the challenges of recruiting patients to RCTs. In this paper, we presented findings from two qualitative process evaluations, which were conducted to inform recruitment to two orthopaedic surgical trials. When considering our findings and those of two recent systematic reviews of the evidence on recruitment to RCTs, we argue that a shift is required in how process evaluations are conducted. We suggest that to optimise recruitment, the wealth of knowledge that has been accumulated is used to inform recruitment and that priority is given to developing less resource-intensive interventions, which use existing evidence to optimise recruitment.

## Supplementary Information

**Additional file 1.** PRESTO Staff Topic Guide. Topic guide used for semi-structured interviews with PRESTO staff.

**Additional file 2.** PRESTO Patient Accepter Topic Guide. Topic guide used for semi-structured interviews with patients who agreed to take part in the PRESTO study.

**Additional file 3.** PRESTO Patient Decliner Topic Guide. Topic guide used for semi-structured interviews with patients who declined to take part in the PRESTO study.

**Additional file 4.** ACTIVE Patient Accepter Topic Guide. Topic guide used for semi-structured interviews with patients who agreed to take part in the ACTIVE trial.

**Additional file 5.** ACTIVE Patient Decliner Topic Guide. Topic guide used for semi-structured interviews with patients who declined to take part in the ACTIVE trial.

**Additional file 6.** ACTIVE Staff Topic Guide. Topic guide used for semi-structured interviews with ACTIVE staff.

## Data Availability

The datasets that we have acquired will not be available as our ethical approval does not permit the sharing of qualitative datasets.

## Abbreviations

ACTIVE: Articular type C pilon fracture Trial comparing Internal Versus External fixation
ED: Emergency department
PRESTO: Pragmatic randomised evaluation of stable thoracolumbar fracture treatment outcomes
QUINTET: Qualitative research integrated within trials
RCT: Randomised controlled trial
UK: United Kingdom
