# Supplementary material for: Embedding qualitative research in randomised controlled trials to improve recruitment: findings from two recruitment optimisation studies of orthopaedic surgical trials
Source: Trials. 2021 Jul 17;22:461. doi: 10.1186/s13063-021-05420-4 (PMC8285860; doi:10.1186/s13063-021-05420-4)
Supplement: Supplementary file 1 — Additional file 1. PRESTO Staff Topic Guide. Topic guide used for semi-structured interviews with PRESTO staff. [file 13063_2021_5420_MOESM1_ESM.pdf]

PRESTO Topic Guide – Staff

At the beginning of all interviews

- The qualitative researcher will introduce themselves to the participant as part of the PRESTO research team.
- The qualitative researcher will explain the PRESTO study and the purposes of the interview
- The qualitative researcher will explain that we would like to audio-record the interview and processes for ensuring anonymity and confidentiality of interview data.
- The qualitative researcher will explain how interview data will be used.
- The qualitative researcher will determine if the participant would like to take part in the study and if so, will obtain verbal and written consent.
- Participants will be provided with the opportunity to ask any questions.

This topic guide summarises the main areas to be explored for each interview. As with any qualitative interview, these headings are intended as a starting point to ensure the primary issues are covered, whilst allowing flexibility for new issues to emerge.

Main interview

- Background information: job role, years of experience.

- What treatment (surgical and non-operative) is currently used for thoracolumbar fractures?
- What treatment do you normally provide or recommend for thoracolumbar fractures?
- Do you normally operate on patients with thoracolumbar fractures?
- What sorts of things influence your decision to recommend surgery/non-operative management?
- Do you have any preferences for how thoracolumbar fractures should be treated?

We would like to understand how you found taking part in the PRESTO study and would like to get your views on the information that was given to patients and the trial processes.

- How did you find being involved in the PRESTO study?
- Are there any aspects of the research that you found difficult?
- Are there any aspects of the study that could be improved to make it better for you?
- How did you find the information that was given to patients
    - Prompt: information on recovery, consent, information sheet.

We would also like to know what you think the potential barriers and facilitators to running a full trial to assess the effectiveness of surgical vs. non-operative management of thoracolumbar fractures would be?

- Prompt if necessary on issues such as: recruitment, consent, retention, identifying sites, surgeon/patient willingness to take part?
- What do you think is a good outcome from intervention for someone with thoracolumbar fracture?
    - What sort of things would indicate the likelihood of a good/bad outcome
- What do you think about the outcome measures used in this study?
    - What different ones would you use?
- What do you think about the idea that depending on the findings of a full scale trial, practice and treatment for thoracolumbar fractures could change?

- Can you tell me what you understand when I say that 'clinical trials often involve a process of participant randomisation'? have you heard of randomisation before?
- What do you think about it – how do you feel about the fact that chance/toss of a coin might affect what treatment patients receive? How important is understanding equipoise in making randomisation acceptable?

- Have you been involved in other clinical studies?
- Would you take part in a research study again?
- Are there any other issues or questions that you would like to raise?

End of interview

- Thank participant and ask if they have any comments
- Explain again about how data will be used and reiterate about anonymity and confidentiality
- Provide opportunity for questions and state that the lead researcher is contactable after the interview, should questions arise.
