# Supplementary material for: Embedding qualitative research in randomised controlled trials to improve recruitment: findings from two recruitment optimisation studies of orthopaedic surgical trials
Source: Trials. 2021 Jul 17;22:461. doi: 10.1186/s13063-021-05420-4 (PMC8285860; doi:10.1186/s13063-021-05420-4)
Supplement: Supplementary file 4 — Additional file 4. ACTIVE Patient Accepter Topic Guide. Topic guide used for semi-structured interviews with patients who agreed to take part in the ACTIVE trial. [file 13063_2021_5420_MOESM4_ESM.pdf]

ACTIVE Topic Guide –

Interviews for patients who are taking part in the Articular Pilon Fracture Trial (ACTIVE)

At the beginning of all interviews:

- The qualitative researcher will introduce themselves to the participant as part of the ACTIVE research team.
- The qualitative researcher will explain the ACTIVE study and the purposes of the interview.
- The qualitative research will explain that we would like to audio-record the interview and processes for ensuring anonymity and confidentiality of interview data.
- The qualitative researcher will explain how interview data will be used.
- The qualitative researcher will determine if the participant would like to take part in the study and If so, will obtain verbal and written consent.
- Participants will be provided with the opportunity to ask any questions and will be assured that they can stop or take a break at any point during the interview.

This topic guide summarises the main areas to be explored for each interview. As with any qualitative interviews, these headings are intended as a starting point to ensure the primary issues are covered, whilst allowing flexibility for new issues to emerge.

Main interview:

- General well-being, family circumstances, occupation, sport/other activities that may be affected by injury?

- Can you tell me a little bit about how you fractured your ankle? (when, how did it happen?)
- Do you think the fracture will have any long term consequences?
- How long do you think it will take you to recover? Do you think you will make a complete recovery?
- Do you think you will be left with any 'disability'? Are you concerned about doing anything in the future? Do you think the fracture will change the sort of things that you do?

- Can you tell me about the treatment you have received so far? Initial presentation; x-ray and ‘diagnosis’; internal/external fixation; rehab [initial / on-going].

- Before the study started did you have any preference for which treatment you were given? If so why?
- Can you tell me about how you were told about the different treatment options available to you? Who told you, how? What information did you receive?
- What did you already know? Were you surprised by any aspect of your care? Have you experienced any difficulties so far?
- What do you think the benefits and difficulties of the treatment you received are? What do you think are the benefits difficulties of the other treatment?
- Given the choice in the future would you have the same/different treatment? If so why?
- Is there anything you would change about the treatment you received?
- Would you say that you are satisfied with your treatment?

- Before this study what did you know about clinical research? Have you been involved previously? Awareness from the media? Previous thoughts about the purpose of clinical research and its value?

- How have you found being involved in the ACTIVE trial?
- Was it something that you really wanted to do?
- Can you tell me about why you decided to get involved in the trial?
- Can you tell me anything about the treatments in the study that you did not have?
- What did you think about the processes for entering the study?
    - Prompt re. experiences of consent, information leaflets, information provided, explanations?
- Is there anything we could do to make the process for entering the study better or clearer?
    - Was there anything you think we didn’t explain or that wasn’t included?
- Are there any aspects of the study that you have found difficult?

- Has anyone explained the follow-up process?
    - Prompt when/how they will get follow-up contact, importance of completion of follow-up, barriers and facilitators to attrition.

- What would make participating worthwhile?
- What do you think will affect your ability to engage with/keep participating in the trial?

- Can you tell me what you understand when I say that clinical trials often involve a process of participant randomisation? Have you heard about randomisation before? What do you think about it – how do you feel that chance/toss of a coin might affect what treatment you are going to get. How important is understanding *equipoise* i.e. whether one treatment is better than another in making randomisation acceptable?
- Can you tell me how you were told about randomisation?
- Would you take part in a research study again?
- How do you think the study has impacted you, have you enjoyed it? Learnt anything?

- Are there any other issues or questions you would like to ask?

End the interview:

- Thank participant ask if they have any other comments
- Explain again about how data will be used and reiterate about anonymity and confidentiality
- Provide opportunity for questions and states that the lead researcher is contactable after the interview should questions arise.
