# Supplementary material for: Embedding qualitative research in randomised controlled trials to improve recruitment: findings from two recruitment optimisation studies of orthopaedic surgical trials
Source: Trials. 2021 Jul 17;22:461. doi: 10.1186/s13063-021-05420-4 (PMC8285860; doi:10.1186/s13063-021-05420-4)
Supplement: Supplementary file 6 — Additional file 6. ACTIVE Staff Topic Guide. Topic guide used for semi-structured interviews with ACTIVE staff. [file 13063_2021_5420_MOESM6_ESM.pdf]

ACTIVE Topic Guide –

Interviews for surgeons and trial recruiters of the Articular Pilon Fracture Trial (ACTIVE)

At the beginning of all interviews:

- The qualitative researcher will introduce themselves to the participant as part of the ACTIVE research team.
- The qualitative researcher will explain the ACTIVE study and the purposes of the interview.
- The qualitative researcher will explain that we would like to audio-record the interview and processes for ensuring anonymity and confidentiality of interview data.
- The qualitative researcher will explain how interview data will be used.
- The qualitative researcher will determine if the participant would like to take part in the study and If so, will obtain verbal and written consent.
- Participants will be provided with the opportunity to ask any questions.

This topic guide summarises the main areas to be explored for each interview. As with any qualitative interviews, these headings are intended as a starting point to ensure the primary issues are covered, whilst allowing flexibility for new issues to emerge.

Main interview:

- Background information (job role, years experience, do you normally operate on patients with closed PILON fractures)
- In your routine work what treatment do you normally provide or recommend for closed PILON fractures? What sorts of things influence your decision to recommend internal/external fixation or non-operative treatment?
- Do you have any preference for how closed PILON fractures should be treated?

We would like to get your opinion about the recruitment of patients.

- How did you explain to patients about the different treatment options available to them?
- How did you find the recruitment process?
- What sort of things helped with recruitment?
- What hindered it?
- When do you think is the best time to recruit patients to the trial?

- What do you think we could do to improve the process of recruiting patients to the trial? Is there anything we could do to make this clearer? Thoughts on study documentation (information leaflets?)

We would like to get your opinion about obtaining consent from patients.

- How did you find the process of consenting to the ACTIVE trial?
- Was there anything that helped or hindered the consent process?
- Do you think that clinicians are the best people to obtain consent for the trial?
- What do you think we could do to improve the process of obtaining consent from patients?

We would also like to understand how you think we could ensure that participants stay in the trial once they agree to take part?

- What sorts of things do you think would prevent patients from dropping out of the trial?
- What do you think are the likely things that would mean a patient would drop out of the trial?

- Are there any aspects of the research that you have found difficult?
- Are there any aspects of the study that could be improved to make it better for you?
- Have you been involved in any other clinical studies?
- Can you tell me what you understand when I say ‘that clinical trials often involve a process of participant randomisation’? have you heard of randomisation before?
- What do you think about it – how do you feel that *chance/toss of a coin* might affect what treatment patients receive. How important is understanding *equipoise* in making randomisation acceptable.
- Would you take part in a research study again?

- Are there any other issues or questions the participant would like to raise?

End the interview:

- Thank participant ask if they have any other comments
- Explain again about how data will be used and reiterate about anonymity and confidentiality
- Provide opportunity for questions and states that the lead researcher is contactable after the interview should questions arise.
